# Ileal and jejunal Peyer’s patches in buffalo calves: Histomorphological comparison

**DOI:** 10.14202/vetworld.2015.1273-1278

**Published:** 2015-11-05

**Authors:** Kritima Kapoor, Opinder Singh

**Affiliations:** Department of Veterinary Anatomy, Guru Angad Dev Veterinary and Animal Sciences University, Ludhiana, Punjab, India

**Keywords:** histomorphology, ileum, jejunum, lymphoid follicle, Peyer’s patch

## Abstract

**Aim:**

The present study was aimed to elucidate the histomorphology of ileal and jejunal Peyer’s patches in the small intestine of buffalo calves and their structural comparison.

**Materials and Methods:**

The study was conducted on neonatal (n=10) and pre-pubertal (n=10) buffalo calves. The age of the postnatal buffalo calves was estimated by their temporary and permanent dentition.

**Results:**

The study revealed that several layers of oval to elongate elliptical lymphoid follicles were observed in submucosa on the anti-mesenteric side in the ileum of early neonatal calves. However, the follicles at this age, in jejunum were of all shapes present within one layer. The interfollicular space was occupied by the interfollicular tissue, which was diffuse and wider around jejunal lymphoid follicles as compared to ileal lymphoid follicles. However, toward the pubertal stage, the number of layers of lymphoid follicles was reduced in ileum due to involution while it remained similar in number in jejunum at this stage.

**Conclusion:**

The ileal Peyer’s patches were found to have started involution more or less around reaching puberty, whereas the jejunal Peyer’s patches appear to be functional throughout the lifespan of the animal.

## Introduction

The gastrointestinal (GI) tract in mammals, although performs the main role of nutrient absorption in digestion, but it represents a key interface between external environment and internal environment of host body. As alimentary tract has the highest microflora; therefore, it is important to differentiate potentially harmful pathogens from resident normal gut microflora.

In the intestine, the gut-associated lymphoid tissue (GALT) acts to protect the animal and generate an immune response against the foreign antigens entering the alimentary tract. GALT is, therefore, one of the largest lymphoid organs in the body comprising 70% of the body’s lymphoid tissue. Since GI tract contains more antibody producing cells than in spleen and lymph nodes combined, it generates immune response by sampling foreign antigens from the lumen [1,2]. The immunoglobulin-A secreted by the intestine is added directly into the lumen.

It, therefore, becomes very crucial to determine the role of lymphoid tissue in the alimentary tract in terms of mucosal immunity. GALT is present throughout the GI tract in mammals as aggregated and solitary lymphoid nodules, known as Peyer’s patches [3,4]. However, the appearance, number and organization of lymphoid follicles differ within small intestine, i.e., between jejunum and ileum. Therefore, in this study, the GALT of ileum and jejunum is compared in neonatal and prepubertal buffalo calves.

## Materials and Methods

### Ethical approval

This study was conducted after approval by the Research Committee and Institutional Animal Ethics Committee.

### Animals

The present study was conducted on neonatal (n=10) and pre-pubertal (n=10) buffalo calves. The age of the postnatal buffalo calves was estimated by their temporary and permanent dentition. The intestinal pieces were collected from them and washed with phosphate buffer saline (pH=7.2) to remove intestinal contents.

### Experimental design

The tissue pieces were fixed in 10% neutral buffered formalin. After fixation was achieved, they were opened along the mesenteric attachment and intestinal walls were examined closely to trace the outline of the lymphoid tissue in jejunum and ileum. Then, the tissue samples were obtained from part of ileum and jejunum containing lymphoid tissue and processed for paraffin blocks preparation by acetone-benzene schedule [5] and sections of 5-6 µm were obtained on glass slides with rotary microtome. To study the histomorphological details, these paraffin sections were stained with hematoxylin and eosin for routine morphology [5], Masson’s trichome for collagen fibers [5], Gridley’s for reticular fibers [6], Verhoeff’s for elastic fibers [6], Alcian blue for mucosubstances (pH=2.5) and McManus’ periodic acid-Schiff (PAS) method for glycogen [5].

## Results and Discussion

**Figure-1:**
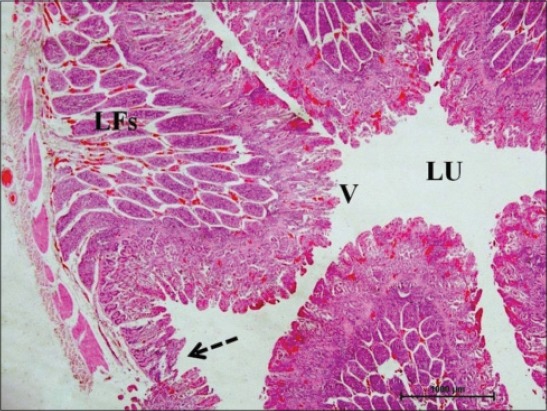
Ileum of 1-month-old buffalo calf showing oval to elongate elliptical lymphoid follicles (LFs) arranged in multiple layers in submucosa on the anti-mesenteric side occupying most of the mucosa, mesenteric side without lymphoid follicles (arrow) surrounding the lumen (Lu) with villi (V) facing toward it (H and E, ×20).

**Figure-2:**
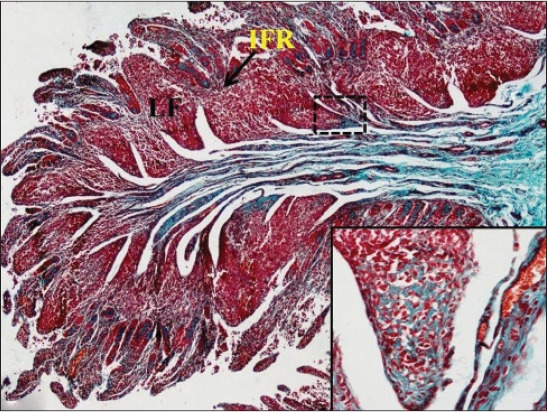
Thick bundle of collagen fibers (dotted arrow) in submucosa encapsulating the lymphoid follicles (LF) from the base toward apex and wide interfollicular region (IFR) in the jejunum of 1-month-old buffalo calf. Masson’s trichrome ×40 (Inset: More collagen fibers toward base of follicles).

**Figure-3:**
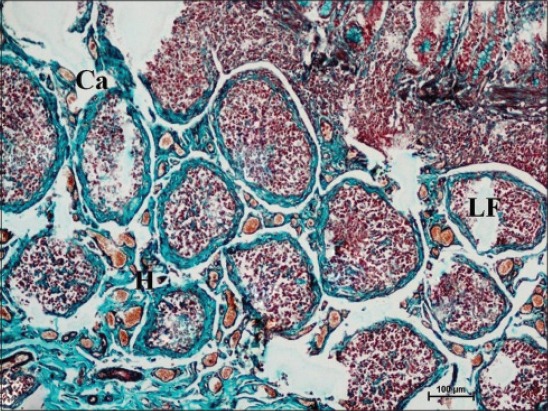
Ileum of 1-month-old buffalo calf showing round lymphoid follicles (LF) surrounded by thick collagen-fiber capsule (Ca) and high endothelial venules (H) in interfollicular space. Masson’s trichrome ×100.

**Figure-4:**
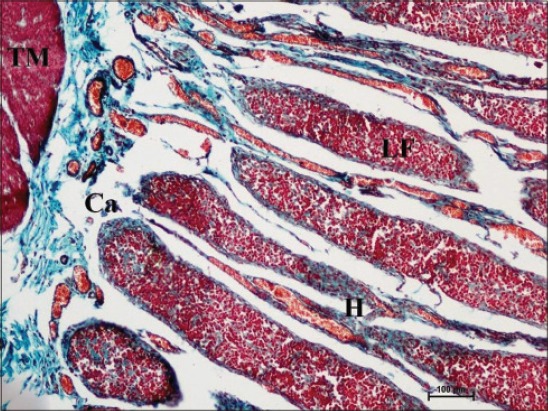
Elliptical lymphoid follicles (LF) present toward serosal side and covered by thick collagen-fiber capsule (Ca), high endothelial venules (H), and tunica muscularis (TM) in photomicrograph of ileum of 1-month-old buffalo calf. Masson’s trichrome ×100.

**Figure-5:**
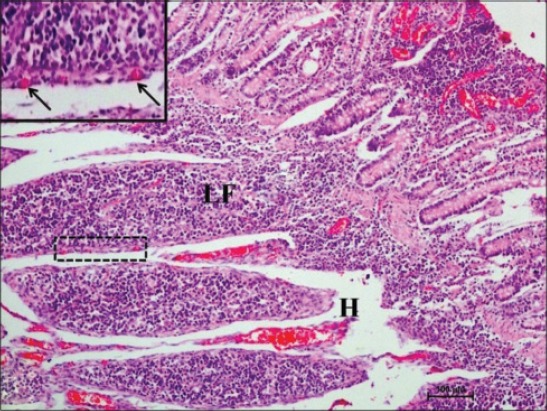
Ileum of 1-month-old buffalo calf showing lymphoid follicle (LF) invading toward epithelium, blood capillaries in the capsule (boxed, arrows), and high endothelial venules (H) in interfollicular space (H and E, ×100).

**Figure-6:**
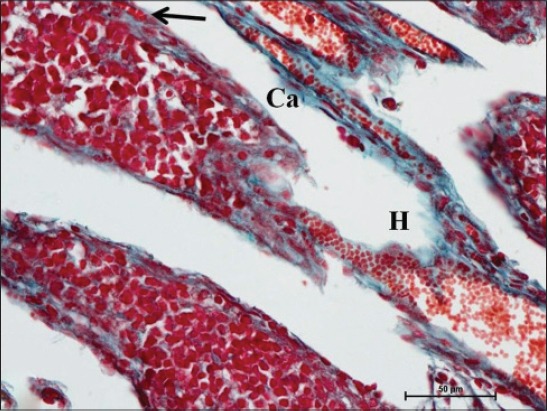
High endothelial venule (H) entering into lymphoid follicles (LF) covered by collagen fiber capsule (Ca) and also blood capillaries within the capsule (arrow) in ileum of buffalo calf at the age of 1 month. Masson’s trichrome ×400.

**Figure-7:**
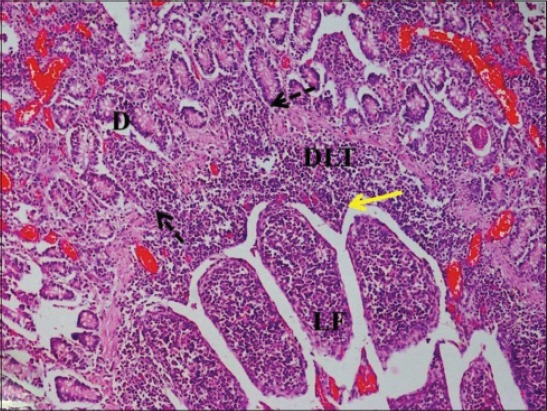
Ileum of 1-month-old buffalo calf showing diffuse lymphoid tissue (DLT) forming dome (D) by disintegration of lamina muscularis mucosae (arrow) and arcs (yellow arrow) above the upper layer of lymphoid follicles (LF) (H and E, ×100).

**Figure-8:**
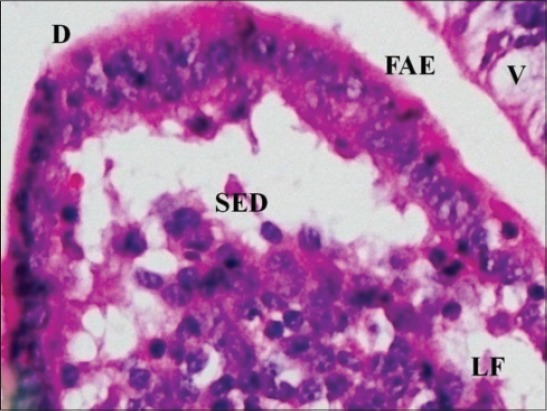
Ileum of 1-month-old buffalo calf showing follicle-associated epithelium (FAE) lining dome (D) and subepithelial dome (SED) of invading lymphoid follicle (LF) surrounded by villi (V) on either sides (H and E, ×400).

**Figure-9:**
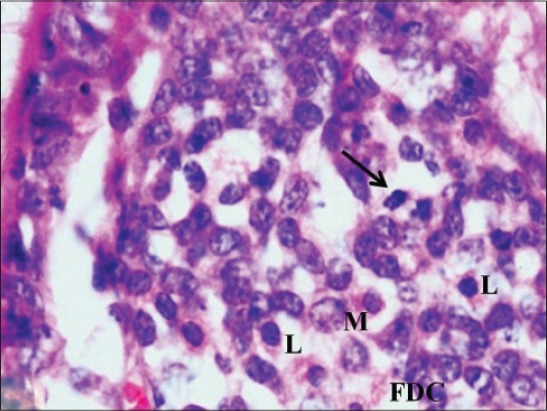
Ileum of 1-month-old buffalo calf showing subepithelial dome region having abundant lymphocytes (L), macrophages (M), follicular dendritic cells (FDC) and mitotic figures (arrow) (H and E, ×1000).

In early neonatal calves, lymphoid follicles were observed in submucosa on the anti-mesenteric side. Shape of the follicles was oval to elongate elliptical in ileum. Moreover, the follicles were arranged in multiple layers in ileum (Figure-1). Similar multiple layered arrangements of ileal lymphoid follicles were observed in full-term buffalo fetuses [7]. In 2-day-old gnotobiotic calves and 6-day-old pigs, the lymphoid follicles were cylindrical to egg shaped with immature germinal centers (GC) [8,9] and white, elliptic uplift, slightly protruding structures were observed as Peyer’s patches in ileum of rats [10]. At this age, in jejunum, follicles of all shapes viz. square, oval or elongated, pear-shaped were present within one layer (Figure-2). Many workers also reported a similar finding, i.e., lymphoid follicles of jejunal Peyer’s patches were oval or rectangular or pear-shaped, situated on the anti-mesenteric border in ruminants [11] and in mouse deer [12]. However, it was also reported in adult river buffaloes that the jejunal Peyer’s patches were randomly distributed at mesenteric as well as antimesentric regions, i.e., their distribution do not follow any specific pattern [13]. In postnatal period at the age of 1-month, dense connective tissue capsule encapsulated the follicles in the lower layer of follicles that were both round and elliptical in ileum (Figures-3 and -4). Abundant blood vessels were present in the capsule of the follicles also (Figures-5 and -6). However, in 2-day-old gnotobiotic calves and 6-day-old pigs a thin sheet of connective tissue capsule encapsulated the lymphoid follicles [8]. In jejunum at this age, the base of the follicles were covered by dense fibrous covering of collagen fibers that joined the connective tissue strands from deep submucosa (Figure-2); abundant reticular fibers also formed framework of lymphoid follicles. At this stage in ileum, prominent domes were observed and also the diffuse lymphoid tissue was found to invade the epithelium at some places forming the dome and propria nodules (Figure-7). The elevated dome regions with non-villous epithelium overlaid the follicles in koala, brushtail possums and ringtail possums [14]. However, a subepithelial dome (SED) region was also observed in protruding lymphoid follicles of ileum just below the dome (Figure-8). SED region, i.e. above the GC region contained developing lymphocytes, macrophages, plasma cells and follicular dendritic cells at this stage (Figure-9). In neonates, at 1 month of age, diffuse lymphoid tissue in the interfollicular region formed arches just above the layer of lymphoid follicles with their concavity facing toward multiple layers of lymphoid follicles (Figure-7) in ileum. Similarly, in 3-month-old buffalo calves, poorly developed interfollicular areas in ileum were comprised of only a very small triangular area and some interfollicular areas were situated below the muscularis mucosae at the apex of the follicles [15]. The junction of arches extended into the interfollicular region and formed interfollicular lymphoid tissue (Figure-7). However, the interfollicular region was wider in jejunum at this stage as compared to ileum (Figure-2). A similar finding was reported in 1-1.5 months old pigs [16] and in red deer [17]. Lymph vessels extended from these arches in interfollicular space. Similar observations were reported in Indian buffalo calves [18]. Others researchers also observed the presence of lymphatic sinuses and interruptions at the subepithelial lymphatic vessels of the tunica mucosa and interfollicular area in rabbit and sheep, respectively [19,20]. Furthermore, abundant high endothelial venules (HEV) and few connective tissue fibers were present within the spaces (Figures-5 and -6). The HEV were also present in the Peyer’s patches in the gut of humans [21]. The intestinal glands in both ileum and jejunum were strongly positive for acidic mucopolysaccharides, whereas capsule and center of lymphoid follicle were weakly positive. Similarly, in chicken that goblet cells of villous epithelium were PAS positive, whereas absent in lymphoepithelium of Peyer’s patches [22]. The follicle-associated epithelium (FAE) over dome was devoid of goblet cells in ileum, therefore, surface epithelial lining of dome was weakly positive for acidic mucopolysaccharides as compared with that of villous epithelium. However, the villous epithelium just above the dome had strong activity for both acidic and neutral mucopolysaccharides. FAE was devoid of goblet cells in sheep [23].

**Figure-10:**
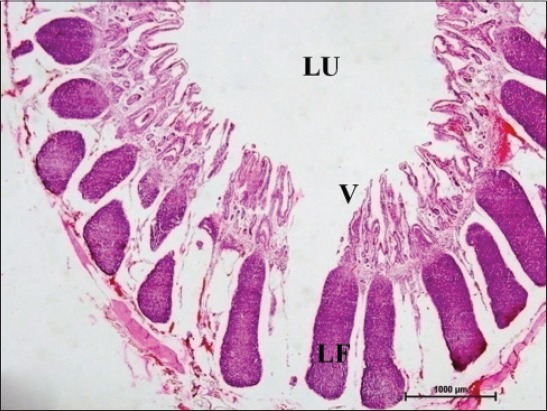
Ileum of 6-month-old buffalo calf showing lymphoid follicles (LF) arranged in 1 or 2 layers, lumen (LU) and villi (V) (H and E, ×20).

**Figure-11:**
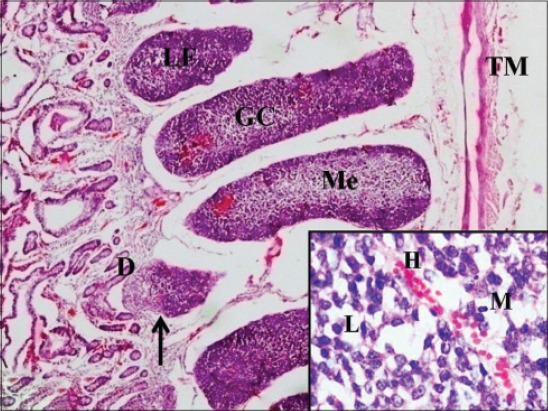
Section same as above showing the presence of germinal center (GC) in lymphoid follicles (LF) with outer dark cortex (C) and inner lighter medulla (Me), involution of lymphoid follicles (arrow) having only the dome (D) and tunica muscularis (TM) (H and E, ×40) (Inset: Medulla showing high endothelial venule (H), lymphoccytes (L), and macrophage (M)).

**Figure-12:**
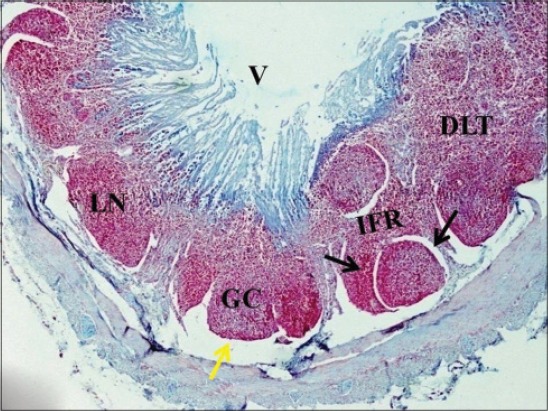
Jejunum of 6-month-old buffalo calf showing large diffuse lymphoid tissue (DLT) in the interfollicular region (IFR) surrounding the solitary lymphoid nodules (LN). The lymphoid nodule is surrounded on its lateral sides by lateral lymph sinus (black arrow) and basal lymph sinus (yellow arrow) toward serosal side (alcian blue ×40).

In 6-month-old calves, the number of layers of elliptical lymphoid follicle in ileum reduced to 1 or 2 (Figure-10). Only the ileal Peyer’s patch regressed to small scattered follicles in older pigs [24] and the reduction in size of ileal follicles was observed in adult dogs [25] and in Bactrian camel [26] also, after 5 years of age. Similarly in red deer, from 6 months of age, the ileal Peyer’s patch follicles began to round up, the interfollicular area in ileal Peyer’s patch also increased and resembled the morphology of the jejunal Peyer’s patch [17]. At this age, jejunal follicles were also seen in single layer with round, square and elliptical shaped follicles protruding in epithelium. At 6 months of age in ileum and in jejunum, the capsule was thinner on developed follicles toward mucosa. The lymphoid follicles were encapsulated by a thin connective tissue capsule made up of reticulin fibers in ileum of dog [25]. By the age of 6 months, developed follicles had clearly visible outer dark cortex and inner lighter area that formed medulla, i.e. GC. Cortex was stained dark due to the presence of tightly packed mature lymphocytes, and few lymphoblasts, medium-sized lymphocytes, macrophages and reticular cells were observed in the medulla (Figure-11). The GC in the patch was large, consisted of light and dark zones in mouse [27] and also each lymphatic nodule showed a peripheral cortex and inner medulla, the GC in buffaloes [28]. However, in day old goat neonates Peyer’s patch revealed secondary nodules with a predominance of lymphocyte, plasma cells, macrophages, reticular cells, red blood cells, etc. [29]. Abundant collagen fibers were present within the GC of the follicle and also in its capsule. At 6 months of age, in ileum, only the dome of the mature follicles remained, and the lower part had started involution (Figure-11). Most of the upper layers of follicles were comprised of dome only with shrinked lower part of the follicle. In jejunum of 6-month-old calves, large areas of diffuse lymphoid tissue surrounded the solitary lymphoid nodules in the form of concavities that formed arches (Figure-12). In lesser mouse deer jejunal Peyer’s patches lymphatic follicles were separated by large interfollicular regions [12]. There were not many changes observed in the arrangement of jejunal Peyer’s patches with increasing age. Similar finding was made in 6-month-old red deer [17]. The villous epithelium in jejunum was reduced due to epithelial degeneration at this stage; therefore few cells were strongly positive for acidic mucopolysaccharides, whereas center of lymphoid nodules was weakly positive for it (Figure-12). Therefore, according to these observations, the ileal Peyer’s patches were found to have started involution more or less around reaching puberty, whereas the jejunal Peyer’s patches function throughout the life to generate an immune response against the invading antigens in the body. Similar findings were observed in sheep [30], alpacas [31], and humans [32].

## Conclusion

The present study suggests that the ileal Peyer’s patches were found to have started involution more or less around reaching puberty, whereas the jejunal Peyer’s patches appear to be functional throughout the lifespan of the animal.

## Authors’ Contributions

KK has planned and designed the study. The collection of samples and laboratory work was done by KK. OS analyzed the data and provided technical support. The manuscript was prepared under the guidance of OS. Both authors participated in draft and revision of the manuscript. Both authors read and approved the final manuscript.
